# Evidence that tirzepatide protects against diabetes-related cardiac damages

**DOI:** 10.1186/s12933-024-02203-4

**Published:** 2024-03-30

**Authors:** Fatemeh Taktaz, Lucia Scisciola, Rosaria Anna Fontanella, Ada Pesapane, Puja Ghosh, Martina Franzese, Giovanni Tortorella, Armando Puocci, Eduardo Sommella, Giuseppe Signoriello, Fabiola Olivieri, Michelangela Barbieri, Giuseppe Paolisso

**Affiliations:** 1 Department of Advanced Medical and Surgical Sciences, University of Campania ‘‘Luigi Vanvitelli’’, P.zza L. Miraglia, 2, 80138 Naples, Italy; 2 Department of Pharmacy, University of Salerno, Fisciano, SA Italy; 3 Department of Mental Health and Public Medicine, Section of Statistic, University of Campania, Naples, Italy; 4 Department of Clinical and Molecular Sciences, DISCLIMO, Università Politecnica delle Marche, Ancona, Italy; 5 Center of Clinical Pathology and Innovative Therapy, IRCCS INRCA, Ancona, Italy; 6 UniCamillus, International Medical University, Rome, Italy

**Keywords:** Tirzepatide, Heart failure, AC16 cell line, High glucose, GIP receptor, GLP-1 receptor.

## Abstract

**Background:**

Glucagon-like peptide-1 receptor agonists (GLP-1RAs) are effective antidiabetic drugs with potential cardiovascular benefits. Despite their well-established role in reducing the risk of major adverse cardiovascular events (MACE), their impact on heart failure (HF) remains unclear. Therefore, our study examined the cardioprotective effects of tirzepatide (TZT), a novel glucose-dependent insulinotropic polypeptide (GIP) and glucagon-like peptide 1 (GLP-1) receptor agonist.

**Methods:**

A three-steps approach was designed: (i) Meta-analysis investigation with the primary objective of assessing major adverse cardiovascular events (MACE) occurrence from major randomized clinical trials.; (ii) TZT effects on a human cardiac AC16 cell line exposed to normal (5 mM) and high (33 mM) glucose concentrations for 7 days. The gene expression and protein levels of primary markers related to cardiac fibrosis, hypertrophy, and calcium modulation were evaluated. (iii) In silico data from bioinformatic analyses for generating an interaction map that delineates the potential mechanism of action of TZT.

**Results:**

Meta-analysis showed a reduced risk for MACE events by TZT therapy (HR was 0.59 (95% CI 0.40–0.79, Heterogeneity: r^2^ = 0.01, I^2^ = 23.45%, H^2^ = 1.31). In the human AC16 cardiac cell line treatment with 100 nM TZT contrasted high glucose (HG) levels increase in the expression of markers associated with fibrosis, hypertrophy, and cell death (p < 0.05 for all investigated markers). Bioinformatics analysis confirmed the interaction between the analyzed markers and the associated pathways found in AC16 cells by which TZT affects apoptosis, fibrosis, and contractility, thus reducing the risk of heart failure.

**Conclusion:**

Our findings indicate that TZT has beneficial effects on cardiac cells by positively modulating cardiomyocyte death, fibrosis, and hypertrophy in the presence of high glucose concentrations. This suggests that TZT may reduce the risk of diabetes-related cardiac damage, highlighting its potential as a therapeutic option for heart failure management clinical trials. Our study strongly supports the rationale behind the clinical trials currently underway, the results of which will be further investigated to gain insights into the cardiovascular safety and efficacy of TZT.

**Graphical Abstract:**

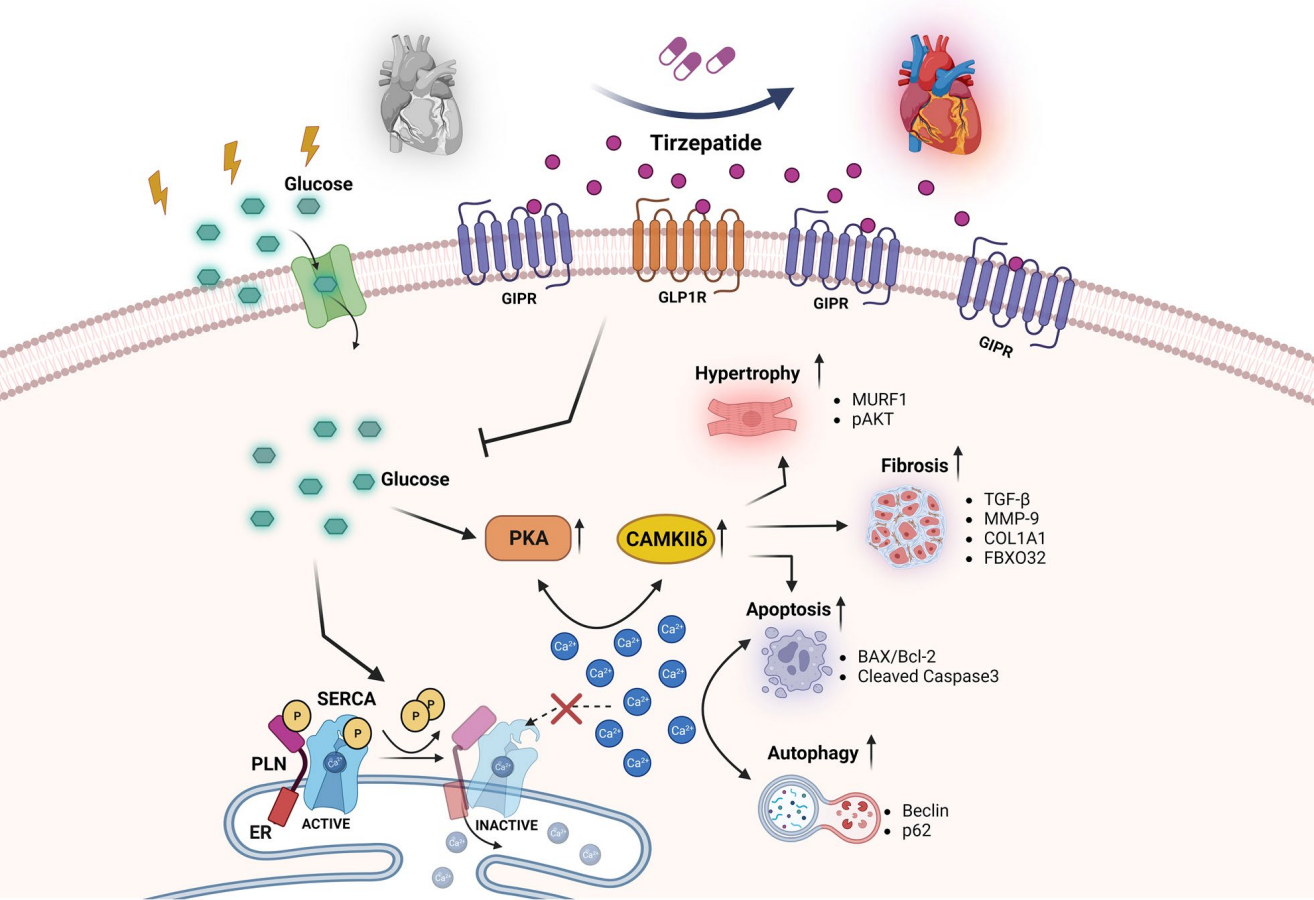

**Supplementary Information:**

The online version contains supplementary material available at 10.1186/s12933-024-02203-4.

## Background

Glucagon-like peptide-1 receptor agonists (GLP-1RAs), widely used antidiabetic drugs, are approved and recommended in several treatment guidelines for reducing the risk of major adverse cardiovascular events (MACE), such as cardiovascular death, non-fatal myocardial infarction (MI), and non-fatal stroke [1–6].

However, unlike sodium-glucose co-transporter 2 (SGLT2) inhibitors [7], evidence of a benefit for GLP-1 RAs in heart failure (HF) is controversial and remains to be fully established in dedicated studies [8].

Several in vitro and in vivo studies have elucidated how GLP-1RAs achieve cardioprotective effects. Firstly, GLP-1RAs indirectly reduce cardiovascular morbidity by lowering glycemia, blood pressure, inflammation, postprandial lipidemia, and body weight [9]. Moreover, the expression of GLP-1R in the human atria, ventricles, and cardiomyocytes suggests a potential direct action of GLP-1RAs on the heart and indicates that the human receptor is expressed in the sinoatrial node [10]. This is in line with the chronotropic effects of GLP-1RAs treatment, which, in a large meta-analysis, was shown to be associated with heart rate increases of up to 3.35 beats/min [8, 11]. Apart from the chronotropic effects, the direct effects of GLP-1, specifically on the heart, have not been comprehensively documented. However, evidence suggests that GLP-1RAs may improve cardiac output and cardiomyocyte survival [9, 12].

Tirzepatide, a novel dual glucose-dependent insulinotropic polypeptide (GIP) and GLP1 receptor (GLP1-1R) agonist has been demonstrated to have cardiovascular safety [13]. Indeed, the SURPASS 4 clinical trial has provided positive findings regarding cardiovascular outcomes in individuals with T2DM. Moreover, ongoing SURMOUNT and SURPASS-CVOT trials could give further insights into the cardiovascular safety of tirzepatide in the future. Early studies of tirzepatide demonstrated more favorable effects on glycemic control, body weight, blood pressure lipid profile, and cardiovascular risk biomarkers compared with GLP-1RAs, supporting the plausibility that tirzepatide may have enhanced cardiovascular efficacy compared with GLP-1RAs [14, 15]. The ongoing SURPASS-CVOT study will definitively assess the CV safety and efficacy of TZT in a cardiovascular outcome trial [16].

So far, we performed a meta-analysis with the primary objective of assessing the occurrence of major adverse cardiovascular events (MACE), incorporating data from randomized clinical trials. Additionally, to investigate the effects of the dual GIP/GLP-1 receptor agonist, TZT, on hypertrophy, fibrosis, calcium handling, and cell death, human cardiac AC16 cell lines were exposed to normal (NG) and high (HG) glucose concentrations for 7 days. Finally, an interactive model, generated through Ingenuity Pathway Analysis to delineate the molecular mechanism by which TZT may ameliorate heart failure, was generated due to the connections among all analyzed markers and the main pathways involved.

## Methods

### Meta-analysis: search strategy, selection criteria, endpoint, and statistical analysis

A systematic literature review was conducted by searching the PubMed database for randomized clinical trials from 2018 to December 2023. The review included 7778 adult patients, regardless of their diabetes mellitus status at baseline, who were assigned to either TZT or placebo/active control. Data from published reports and previous meta-analyses were utilized, and all included manuscripts were manually searched for any additional studies.

Among the studies evaluated, three were found to be eligible, while others were excluded for not meeting eligibility criteria. Data from 7778 patients included in the SURPASS-4 study [17], the SURPASS Clinical Trials Program (which included 7 randomized clinical trials), and SURMOUNT-1 (a study enrolling adult subjects with obesity) [18] were pooled for the meta-analysis, analyzing 7778 patients treated with Tirzepatide and 3971 control patients.

The primary outcome assessed was the Hazard Ratio (HR), measuring the reduction in Major Adverse Cardiovascular Events (MACE) compared to an active control or placebo. The studies reported the estimation of HR and corresponding 95% confidence intervals. A random-effects meta-analysis was conducted, and the index of study heterogeneity (I^2^) indicated low heterogeneity. A forest plot of the meta-analysis was created using Stata software (version 16.0, Stata Corp., College Station, TX).

### Cell culture

AC16 human cardiomyocyte cell lines were purchased from EMD Millipore (cod. SCC109). Following the manufacturer's instructions, the cell line was tested and authenticated for mycoplasma contamination, resulting in negative data. Cells were cultured in Dulbecco's Modified Eagle's Medium /Nutrient Mixture F-12 (cod. D8437, Sigma) containing 12.5% fetal bovine serum (FBS) (cod. ECS0180L, Euroclone), 1% antibiotics penicillin–streptomycin (cod. ECB3001D, Euroclone), and 1% of L-glutamine (cod. ECB3000D, Euroclone). The cell line was maintained in the incubator at 37 °C and 5% CO_2_. The cells were grown between 4 and 6 passages, and experiments were performed in triplicate. AC16 were exposed to 33 mmol/L D glucose (cod. G8644, EMD Millipore) for 7 days [19] and treated with tirzepatide (LY3298176, selleckchem) at a concentration of 100 nM. The medium was changed every 48 h. Normal glucose, NG, was considered the cells exposed to normal glucose concentration (5.5 mmol/L) and cultured for 7 days. A dose–response curve, using cell viability and toxicity assay, was performed to evaluate the right concentration of tirzepatide to carry out experiments (Additional file 1: figure).

### Protein extraction and western blotting

Cells were dissolved in lysis buffer containing protease inhibitors (Tris HCL pH8 10 mM, NaCl 150 mM, NaF 10 mM, NP40 1%, PMSF 1 mM). Then, the proteins were subjected to 10% sodium dodecyl sulfate–polyacrylamide gel electrophoresis (SDS-PAGE) and transferred to 0.22 µm polyvinylidene fluoride (PVDF) membranes. The membranes were blocked with 5% non-fat milk in TBS-T (Tris-buffered pH 8 0.15% Tween 20) at room temperature for 1 h and then incubated with primary antibodies diluted in TBS-T (dilutions according to datasheet). Primary antibodies used to detect proteins were: p62/SQSTM1 (E-AB-70387, elabscience), BECN1 (E-AB-53242, elabscience), BCL2 (E-AB-15522, elabscience), Collagen1 (E-AB-81499, elabscience), TGF beta (ab179695, Abcam), MURF1 (ab183094, Abcam), Fbx32 (ab168372, Abcam), MMP9 (ab76003, Abcam), BAX (ab32503, Abcam), CASP3 (ab32351, Abcam), active CASP3 (E-AB-22115, elabscience), CAMKII delta (PA5-22,168, Thermofisher), phospho-CAMKII (PA5-37,833, Thermofisher), SERCA2 (MA3-919, Thermofisher), phospho-SERCA2 (PA5-117,240, Thermofisher), phospholamban (PLN) (MA3-922, Thermofisher), phospho-phospholamban (p-PLN) (PA5-114,620, Thermofisher), PKA (PA5-17,626, Thermofisher), AKT (2920, Cell signaling), p-AKT (4060, Cell signaling) overnight at 4° C. As an internal control, Vinculin (ab129002, Abcam) and beta-tubulin (ab6046, Abcam) were used for protein expression normalization. After three washes in TBS-T, the membrane was incubated with corresponding secondary antibodies, goat anti-rabbit IgG-h + HRP Conjugated (cod. A120-101P Bethyl) or donkey anti-mouse IgG-h + HRP Conjugated (cod. A90-137P Bethyl) for 1 h at room temperature. Immuno complexes were visualized using Clarity Max Western ECL Substrate (cat. 1705062, Bio-Rad Laboratories) and visualized using ChemiDoc Imaging System with Image Lab Software Version 6.1 software (Bio-Rad Laboratories). The molecular weight of proteins was estimated with prestained protein markers (cod. G623 Opti-Protein-Marker abm). Densitometry analysis was performed using Image J software.

### RNA extraction and quantitative real-time PCR

Total RNA was isolated and purified using miRNeasy Mini Kit (cod. 217,004, Qiagen) according to the manufacturer's instructions for human cell samples. Then cDNA was synthesized from 1 ug of total RNA using QuantiTect Reverse Transcription Kit (cod. 205,310, Qiagen). mRNA levels were determined by qPCR with Green-2 Go qPCR master mix (cod. QPCR004-5 Biobasic) using Rotor-GENE Q (Qiagen).

Primer assays were used to detect gene expression: GIPR: Hs.PT.58.4302958. GLP-1R: Hs.PT.58. 39163702.g. TGF-b: Hs.PT.58.39813975; COL1A1: Hs.PT.58.15517795; CASP3: Hs.PT.56a.25882379.g; MMP9: Hs.PT.58.22814824.g; ATP2A2: Hs.PT.56a.39859858.g; CAMK2D: Hs.PT.56a.25723872.g; PRKACA: Hs.PT.58.2681637; PLN: Hs.PT.58.23189767; BAX: Hs.PT.56a.2333204; BCL2: Hs.PT.56a.2905156; BECN1: Hs.PT.58.504143; NUP62: Hs.PT.58.39408905; FBX32: Hs.PT.58.19947148; MURF1: Hs.PT.58.39092203; GAPDH: Hs.PT.58.39769835; β-actin: fw: 5′—CATCCGCAAAGACCTGTACG—3′, rv: 5′—CCTGCTTGC TGATCCACATC—3′. A threshold cycle (C_t_) value was obtained for each amplification cycle, and Δ_Ct_ was calculated as the C_t_ difference between target mRNA and housekeeping mRNA (β-Actin). The fold increase of mRNA expression compared with NG was calculated using the 2^−ΔΔCt^ method. The histograms reported the genes of 3 separate experiments, where the NG value was set as 1.

GIPR and GLP1R mRNA levels were determined by QIAcuity EG PCR kit (250,112, Qiagen) using QIAcuity One, 5plex (01300, Qiagen). For the Eva Green (EG) protocol, thermal cycling was as follows: one cycle of incubation at 95 °C for 2 min; 40 cycles of 95 °C for 15 s, 60 °C for 15 s, and 72 °C for 15 s; then incubation at 40 °C for 5 min. The reactions were carried out in Nanoplate 26 k (24-well). Results obtained were analyzed using the QIAcuity Software Suite version 2.1.7.182.

### Cell viability assay

Cell viability was assayed by Cell Counting Kit-8 (CCK-8, CK04, Dojindo) according to the manufacturer's protocols. Briefly, AC16 cells were seeded into 96-well plates and treated with HG and tirzepatide for 7 days. After specific treatment, 10 μL of CCK-8 solution was added to each well and incubated for 2 h at 37 °C. The absorbance was then recorded at 450 nm using a microplate reader (Sunrise absorbance reader, TECAN). The relative cell viability was normalized with the control group using optical density values, and three independent experiments were conducted.

### Cytotoxicity LDH assay

LDH release to media was assayed using the Cytotoxicity LDH Assay Kit-WST (CK12, Dojindo). According to the manufacturer’s protocol. Briefly, AC16 cells were seeded into 96-well plates and treated with HG and tirzepatide for 7 days. After specific treatment, 10 μL of lysis buffer solution was added to each well of high control and incubated for 30 min at 37 °C. Next, 100 µl of the working solution was added to all cell wells and incubated for 30 min in a dark place at room temperature. After incubation time, 50 µl of stop solution was added to all cell wells, and the absorbance was then recorded at 490 nm using a microplate reader (Sunrise absorbance reader, TECAN). The relative cell cytotoxicity was normalized with the control group using optical density values and three independent experiments.

### Annexin V-FITC apoptosis detection

Cell death was evaluated using an annexin V-FITC apoptosis staining/detection Kit (ab273273, abcam). Cells were cultured in 6-well plates and exposed to HG and tirzepatide treatment for 7 days. According to the manufacturer's instructions, cells were collected with trypsin, washed with 1X wash buffer, and centrifuged for 5 min at 400 ×*g*. According to the kit protocol suggestion, 1 × 10^5^ cells were resuspended in 500 μL of 1X Binding Buffer, then 5 μl of Annexin V-FITC and 1 μl of SYTOX Green dye added to each sample and for 10 min incubated at room temperature in the dark. Measurements were carried out using BD Accuri C6 Plus Personal Flow Cytometer (BD biosciences) at Ex/Em = 480/530 nm. Data processing was performed using FlowJo BD Accuri C6 Plus software for windows.

### Autophagy detection

According to the manufacturer's protocol, Autophagy Assay Kit was performed in AC16 cells using the Autophagy Assay Kit (ab139484, abcam). Briefly, cells were seeded in 6-well plates and exposed to HG and tirzepatide for 7 days. According to the manufacturer’s instructions, cells were trypsinized, collected with 100 µL of 1 × assay buffer, and centrifugated for 5 min at 400 ×*g*. According to the kit, protocol cells were resuspended in 250 µl of diluted green dye staining solution and incubated for 30 min at 37  C and subsequently washed two times with 1 × assay buffer and resuspended in 1X assay buffer for measurement that were carried out using BD Accuri C6 Plus Personal Flow Cytometer (BD biosciences) with FITC filter Ex/Em = 480/530 nm. Data processing was performed using FlowJo BD Accuri C6 Plus software for windows.

### Cell proliferation: fluorescence-activated cell sorting (FACS)

Cell proliferation assay was performed in AC16 cells using the Ki-67 Alex Fluor 488 Conjugate antibody (11,882, cell signaling). After 7 days of treatment with HG and tirzepatide, cells were trypsinized, collected, and washed gently with PBS, resuspended in 100 µl 4% formaldehyde per 1 million cells and incubated for 15 min at room temperature. After the cell was washed with PBS, permeabilized with 90% methanol ice-cold, and incubated for 10 min on ice. Cells were washed with PBS, resuspended in 100 µl of the primary antibody with 1:50 dilution, and incubated for 1 h at room temperature and dark place. Cells were washed and resuspended in the next step with PBS for measurement that was carried out using BD Accuri C6 Plus Personal Flow Cytometer (BD biosciences) with FITC filter in Ex/Em = 480/530 nm. DData processing was performed using lowJo BD Accuri C6 Plus software for Windows.

### Pathway enrichment analysis

QIAGEN Ingenuity Pathway Analysis (IPA) software (QIAGEN, Milan, Italy) was used for enrichment analysis. The ‘‘core analysis’’ function was used to interpret the data based on biological processes, canonical pathways, and gene networks. Each gene identifier was mapped to its corresponding gene object in the Ingenuity Pathway Knowledge Base (IPKB). The p-value of 0.05 was set as the cutoff value for the enrichment. The top enrichment results of the Molecular and Cellular Function were used as a focus point to connect all the available data using the tools “Connect” and “Path Explorer”.

### Statistical analysis

Results are reported as the means ± SEM. The difference between the mean values was assessed using a one-way analysis of variance (ANOVA) test. Differences between the mean values were considered significant at a p-value of < 0.05. Statistical analyses were performed using SPSS v 26 software (Chicago, IL, USA).

## Results

### Meta-analysis of clinical results

#### Cardioprotective effect of TZT by meta-analysis

**Fig. 1:**
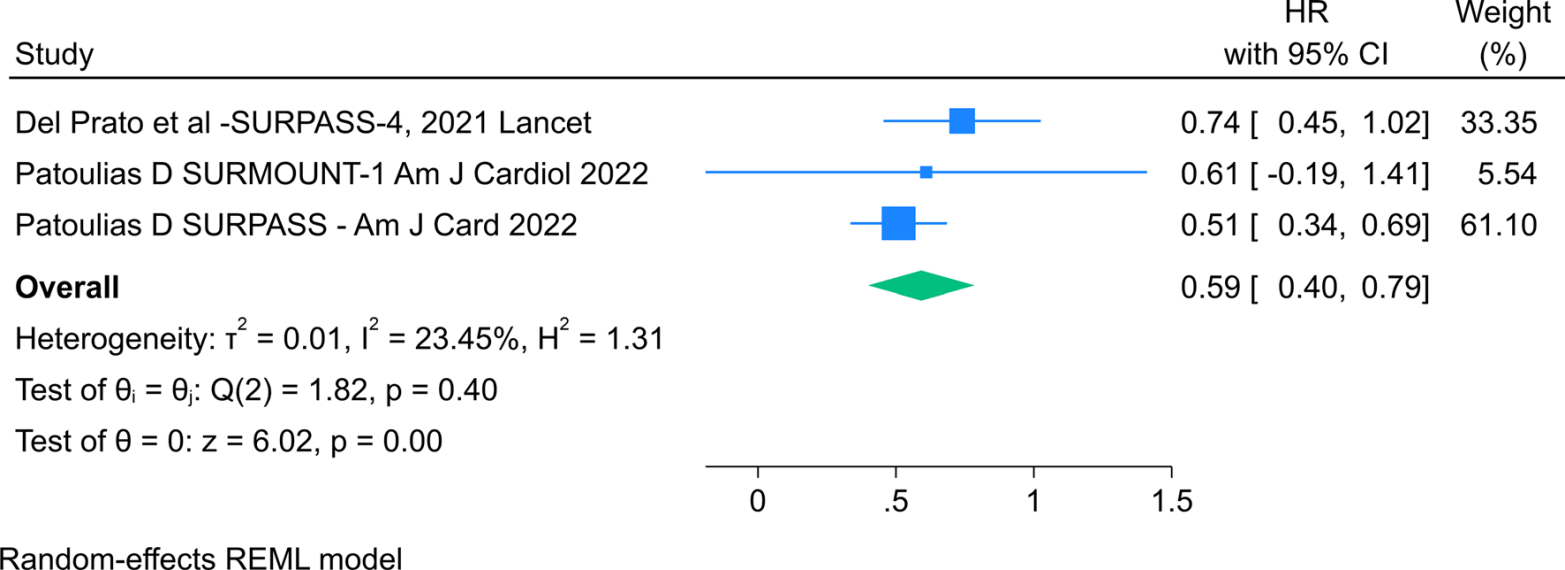
Meta-analysis of Cardiovascular Safety of Tirzepatide in Randomized Clinical Trials (2020–2023) The forest plot of the meta-analysis was created using Stata software (version 16.0, Stata Corp., College Station, TX)

Data from 7778 patients enrolled in the SURPASS-4 study (SURPASS-4 [17]), the SURPASS Clinical Trials Program (which included 7 randomized clinical trials) and SURMOUNT-1 [18] were included in the meta-analysis to evaluate the MACE-4 events, such as cardiovascular death, myocardial infarction, stroke, hospitalization for unstable angina in patients treated with TZT compared to the control group. The estimate of the overall HR was 0.59 (95% CI 0.40–0.79, Heterogeneity: r^2^ = 0.01, I^2^ = 23.45%, H^2^ = 1.31) indicating that TZT resulted in a significant reduction in the risk for a major adverse cardiovascular event (MACE) compared with control (Fig. 1).

### In vitro results in AC16 cell line* results in AC16 cell line*

#### Gene expression of GIPR and GLP1R in AC16 cell line

**Fig. 2:**
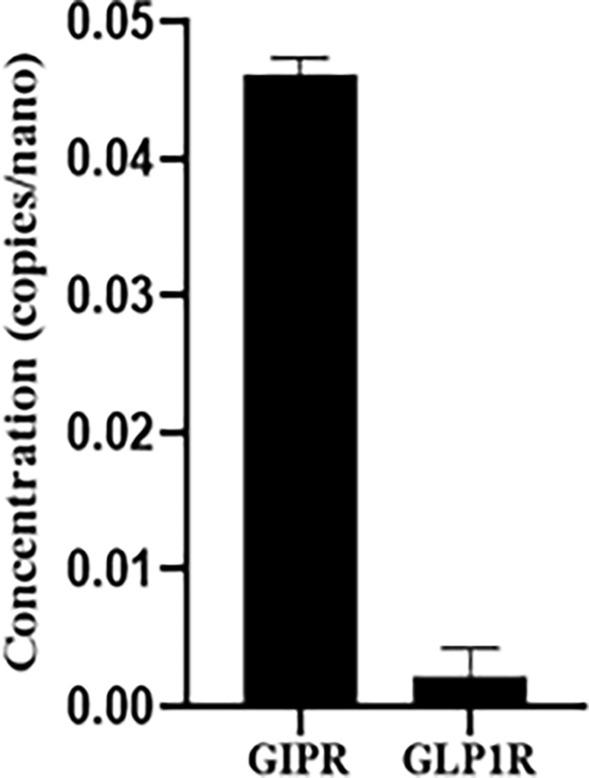
Gene expression of GIPR and GLP1R in AC16 cell line. Gene expression analysis showed a disparity in expression levels between GIPR and GLP1R. Gene expression was normalized to the housekeeping gene GAPDH. Data are presented as mean ± SEM of three independent experiments. p < 0.01

Gene expression analysis demonstrates a significantly higher GIPR expression than GLP1R in the human AC16 cardiac cell line (Fig. 2) (p < 0.01).

### Effects of TZT on cell remodeling induced by fibrosis and hypertrophy

**Fig. 3:**
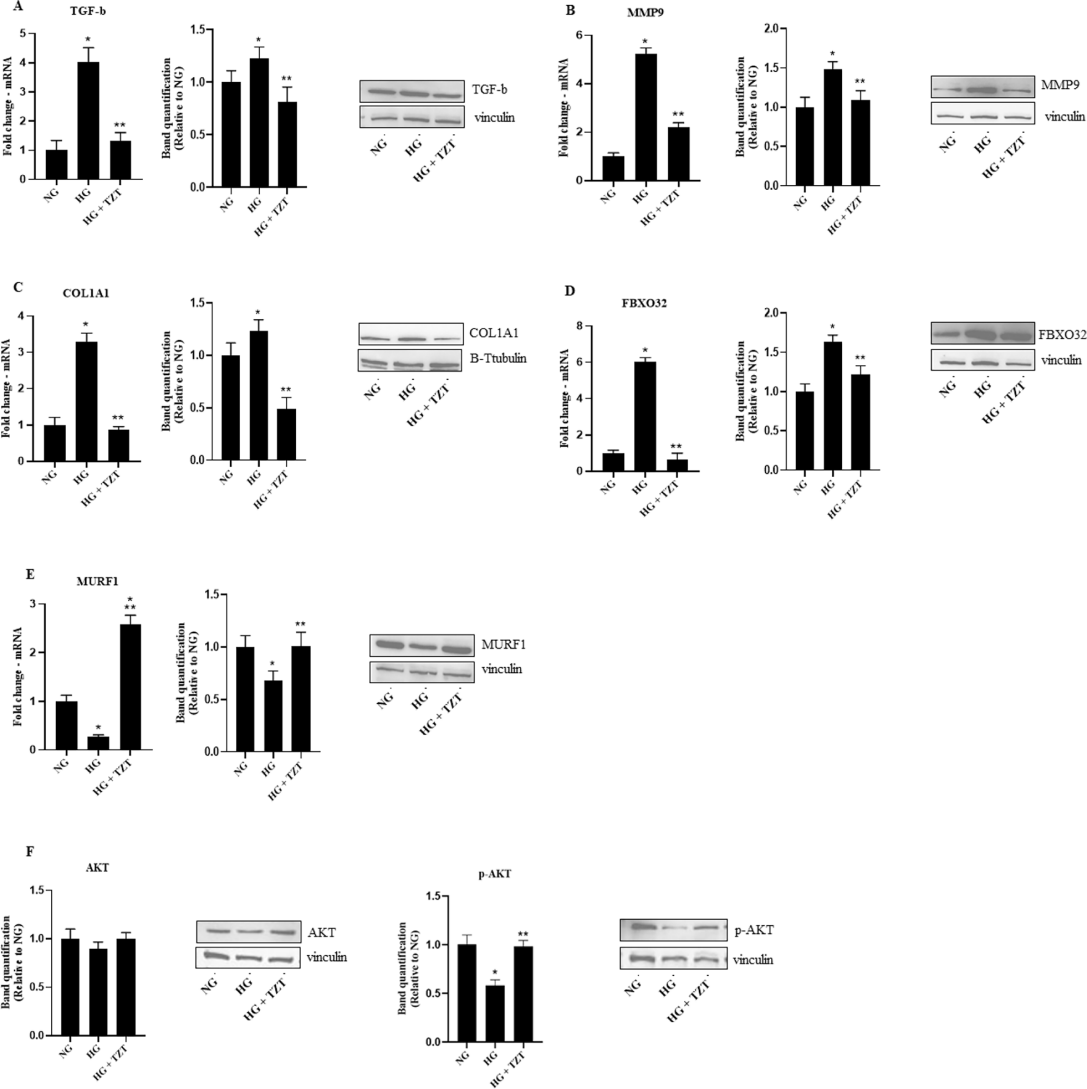
TZT Effects on fibrosis, hypertrophy, and Akt signaling markers mRNA expression and protein levels of **A** TGF-β, **B** MMP9, **C** Collagen, **D** FBXO32, and **E** MURF1 exposure to normal glucose (NG), high glucose (HG), or HG with 100 nM TZT after 7 days. Also, **F** Akt and p-Akt protein levels were shown. β-Actin was used as an internal control in gene expression. The fold increase of mRNA expression compared with NG was calculated using the 2^−ΔΔCt^ method. Protein expression densitometry analysis was performed using Image J 1.52n software. Data are mean ± SEM of three independent experiments. *p < vs NG; ** p < vs HG

High glucose (HG) upregulated fibrosis markers, such as TGF-β (p < 0.009 vs NG), MMP9 (p < 0.05 vs NG), and Collagen (p < 0.04 vs NG) mRNA expression and protein level (p < 0.05 vs NG). In contrast, TZT addition was associated with an opposite trend (p < 0.05 vs. HG) (Fig. 3A, B, C). Similarly, HG-induce upregulation of FBXO32 (p < 0.03 vs NG) and downregulation of MURF1 (p < 0.05 vs NG) mRNA expression and protein levels (p < 0.05 vs. NG for both), while TZT counteracted such negative HG-mediated impact (p < 0.05 vs. HG) (Fig. 3D, E).

Moreover, HG did not induce a statistical reduction in the total form of AKT at the protein level but caused a statistical decrease in the phosphorylated form of AKT (p < 0.05 vs NG). Such an HG-mediated effect was antagonized by TZT (p < 0.05 vs HG) (Fig. 3F).

### Effects of TZT on calcium handling changes

**Fig. 4:**
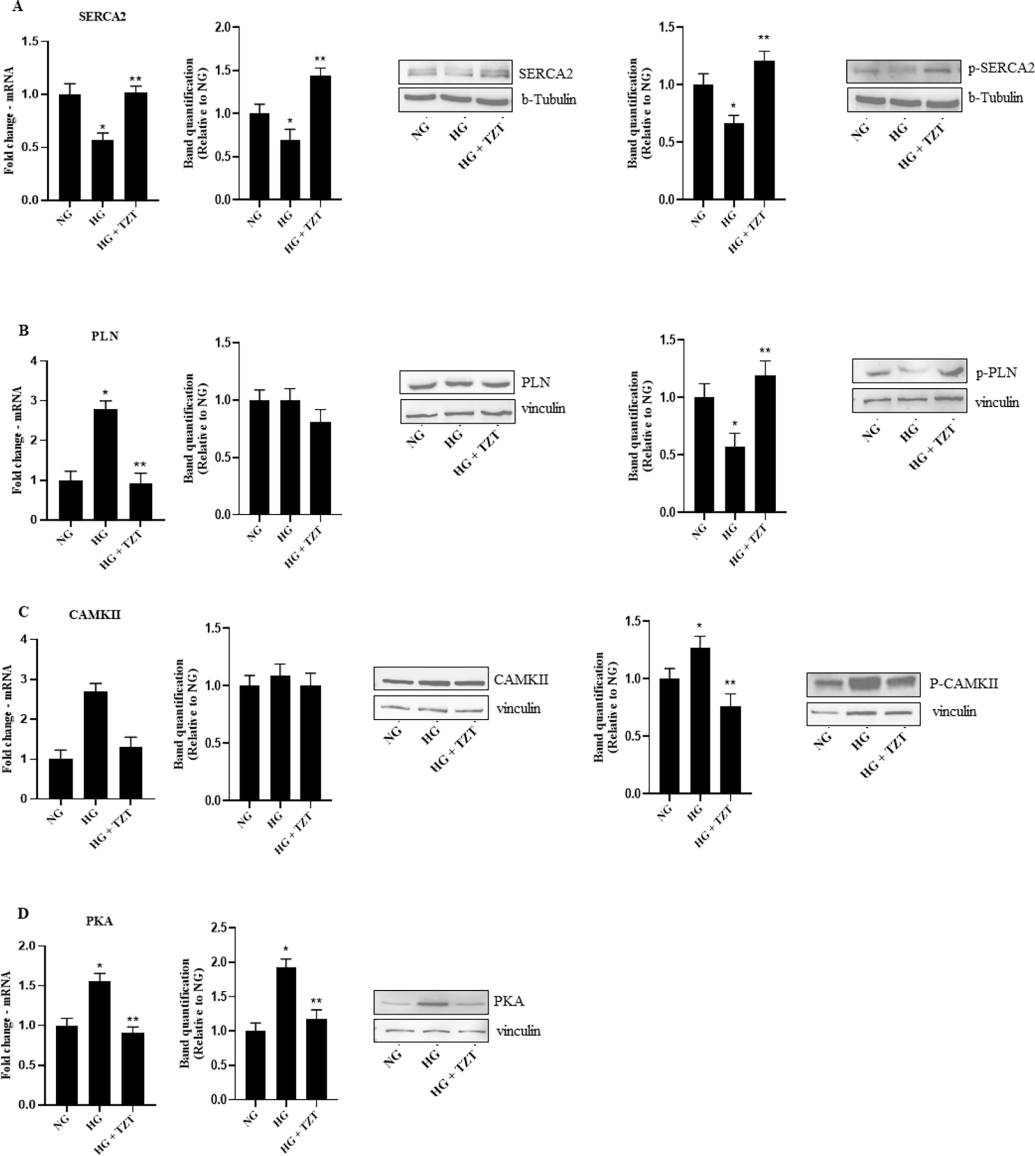
Effects of TZT on calcium homeostasis markers **A** SERCA2 mRNA expression, total protein and phospho-T484 protein levels **B** PLN mRNA expression, total protein and phospho-S16 and -T17 of PLN. **C** CAMKII mRNA expression, total form of CAMKII and phospho-T287 **D** mRNA expression and protein levels of PKA exposure to NG, HG, or HG with 100 nM TZT after 7 days. β-Actin was used as internal control for gene expression. The fold increase of mRNA expression compared with NG was calculated using the 2^−ΔΔCt^ method. Protein expression densitometry analysis was performed using Image J 1.52n software. Data are mean ± SEM of three independent experiments. *p < vs NG; ** p < vs HG

SERCA2 and SERCA2 active form, phosphorylated in threonine 484, at mRNA and protein levels, respectively, were downregulated by HG (p < 0.05 vs NG) while TZT contrasted such effect (p < 0.05 vs HG). (Fig. 4A).

HG showed upregulation of PLN (p < 0.007 vs NG), CAMKII (p < 0.05 vs. NG), and PKA (p < 0.001 vs NG) mRNA expression, with TZT addition associated with an opposite trend (p < 0.05 vs HG) (Fig. 4B, D).

Analyzing the phosphorylated form of CAMKII in threonine 287 protein expression showed its upregulation in HG (P < 0.05 vs NG). In contrast, the cells exposed to HG and treated with TZT showed that TZT antagonized such HG-related upregulation (p < 0.05 vs HG) **(**Fig. 4C).

### Protective Effects of TZT on cell proliferation, viability, and toxicity in AC16 Cells

Effects of TZT on cell viability, proliferation, and toxicity were evaluated in cardiomyocytes AC16 cell line, and they were exposed to HG for seven days.

**Fig. 5:**
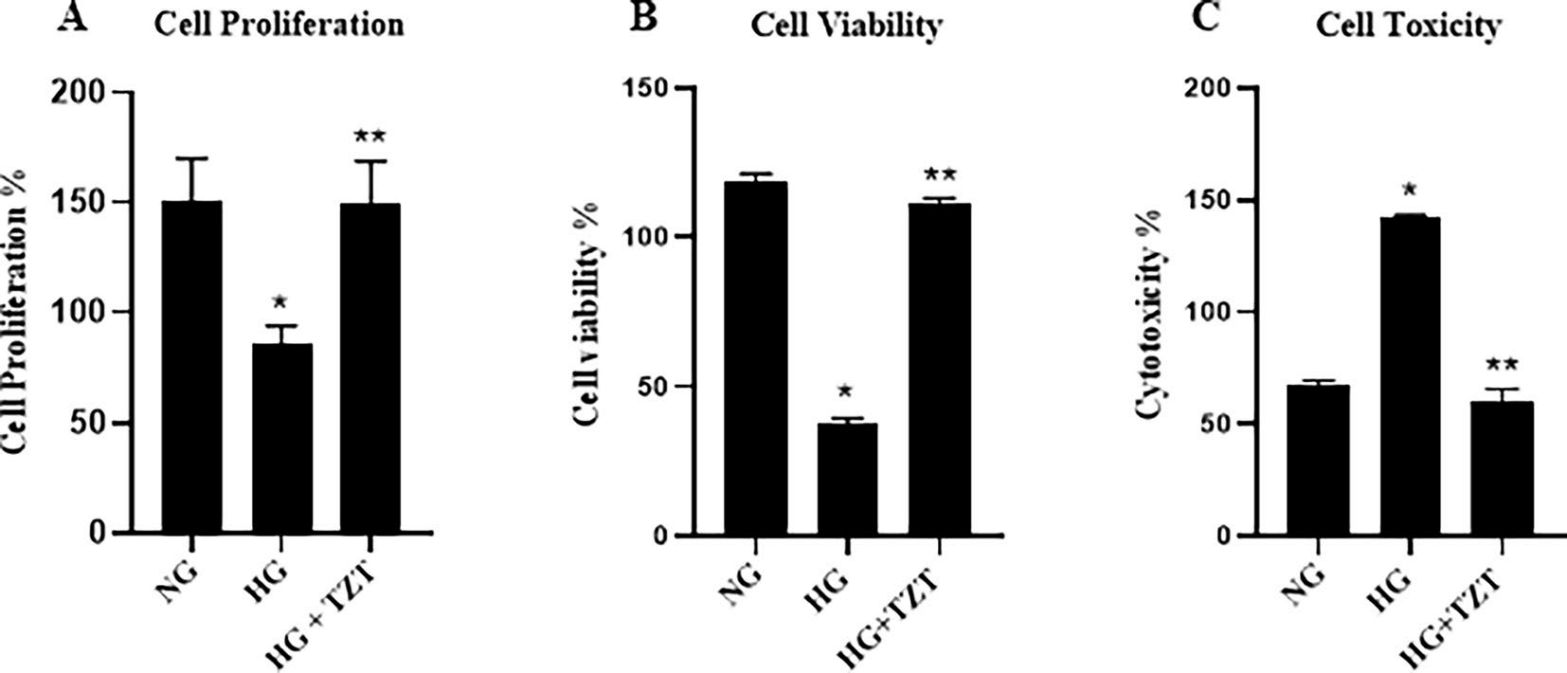
Protective Effects of TZT on cell proliferation, viability, and toxicity **A** AC16 cell proliferation analysis performed after cell staining with Ki-67 marker and the histogram represents median of fluorescence (FITC). **B** Cell viability was assessed using a CCK-8 assay after 7 days of exposure to normal glucose (NG), high glucose (HG), or HG with 100 nM TZT. **(C)** Cell toxicity assay performed with Lactate dehydrogenase (LDH) activity assay. Data are presented as mean ± SEM of three independent experiments. *p < vs NG; ** p < vs HG

HG treatment reduced cell proliferation marker Ki-67 and cell viability percentage and increased LDH level compared to cells exposed to NG concentration (p < 0.001 vs NG for both). The addition of 100 nM of TZT in cells exposed to HG prevented negative HG-mediated impacts on cell viability reduction, proliferation, and high LDH level (p < 0.001 vs. HG for all) (Fig. 5A, B, and C).

### Effects of TZT on cell death

**Fig. 6:**
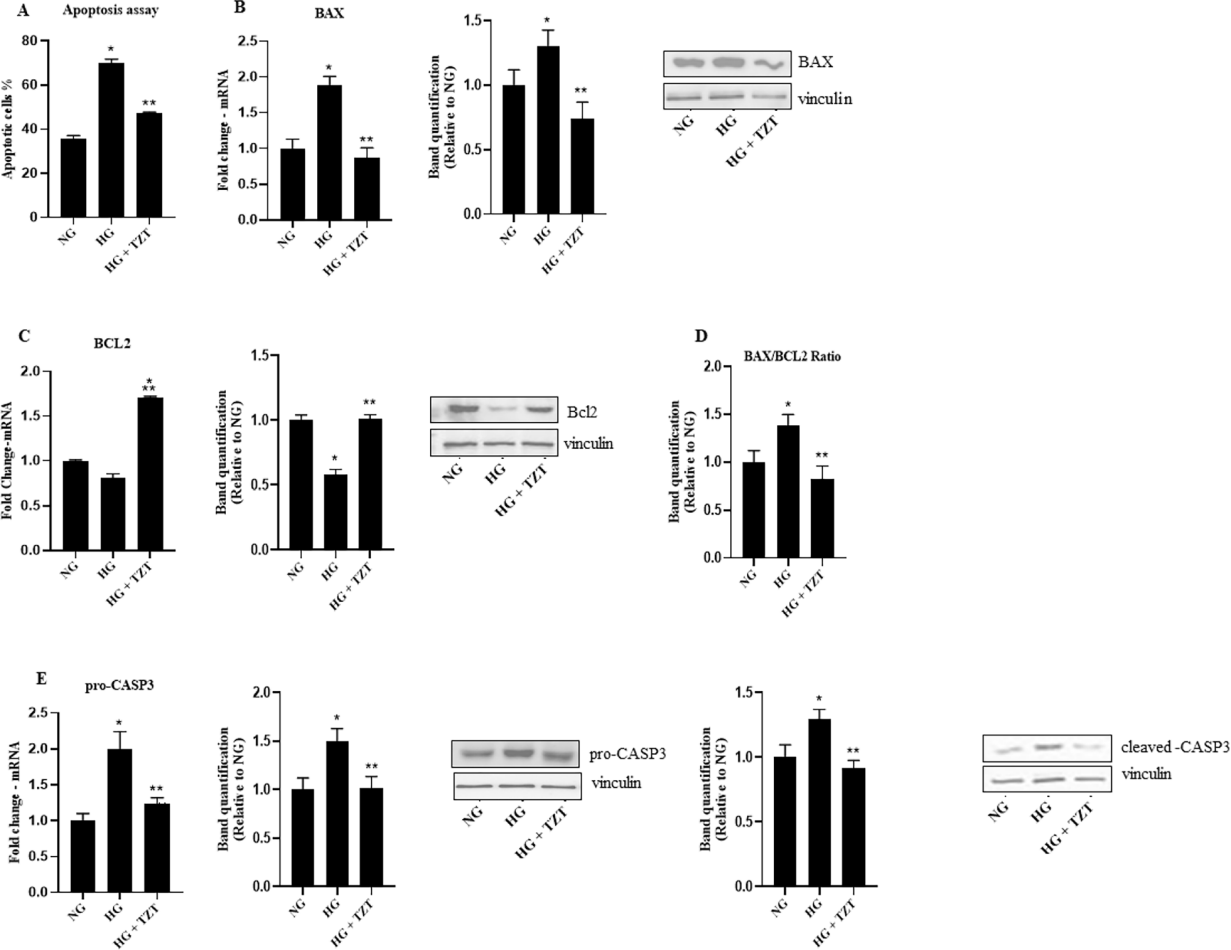
TZT Effects on apoptosis in AC16 cells exposed to high glucose **A** Apoptosis was measured using Annexin V-FITC staining followed by a flow cytometer. The histogram represents the median of fluorescence (FITC). mRNA expression and protein levels of pro-apoptotic **B** BAX, anti-apoptotic **C** Bcl2 and analysis of **D** BAX/Bcl2 ratio at the protein level. Also, mRNA expression and protein levels of **E** Total and cleaved form of caspase-3 exposure to normal glucose (NG), high glucose (HG), or HG with 100 nM TZT were shown. The fold increase of mRNA expression compared with NG was calculated using the 2^−ΔΔCt^ method. Protein expression densitometry analysis was performed using Image J 1.52n software. Data are mean ± SEM of three independent experiments. *p < vs NG; ** p < vs HG

Effects of TZT on apoptosis and autophagy were also evaluated. HG treatment induced an increase in apoptotic cell percentage compared to NG (p < 0.05 vs. NG), while TZT counteracted the HG-induced apoptosis (p < 0.05 vs HG) (Fig. 6A).

Prominent mRNA expression and protein levels of the main genes involved in apoptosis were also quantified. HG treatment increased BAX (p < 0.001 vs. NG) and decreased Bcl2 (p < 0.008 vs NG) mRNA expression and protein levels compared to the NG. In contrast, TZT produced opposite changes to the HG-mediated ones for both protein levels (p < 0.05 vs. HG) (Fig. 6B) but not for Bcl2 mRNA, which resulted unaffected. (Fig. 6C). So far, HG showed an increase in BAX/Bcl2 ratio in protein level (p < 0.05 vs. NG), which was antagonized by TZT presence (p < 0.05 vs HG) (Fig. 6D).

HG-induced upregulation in the total form of CASP3 at mRNA (p < 0.01 vs. NG) and protein levels compared to the NG condition (p < 0.05 vs NG), with TZT opposing such effect. (p < 0.05 vs HG). The protein level of CASP3 cleaved form was increased by HG presence (p < 0.001 vs. NG) (Fig. 6E), but TZT reversed such phenomena.

**Fig. 7:**
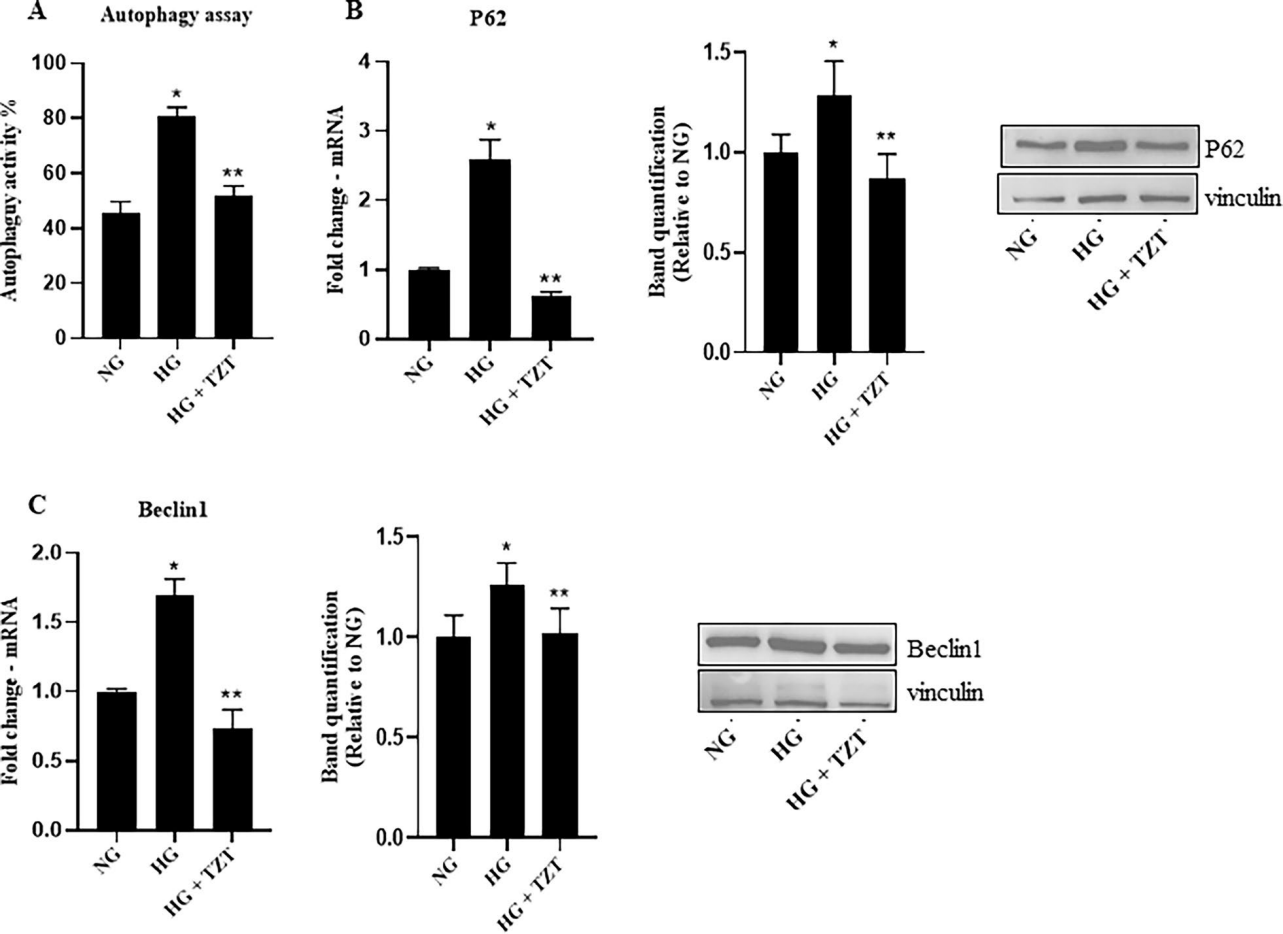
Effects of TZT on autophagy markers **A** Cell autophagy analysis was performed by flow cytometer. The histogram represents median of fluorescence (FITC). mRNA expression and protein levels of the autophagy marker **B** p62 and **C** Beclin1 exposure to normal glucose (NG), high glucose (HG), or HG with 100 nM TZT after 7 days. β-Actin was used as internal control for gene expression. The fold increase of mRNA expression compared with NG was calculated using the 2^−ΔΔCt^ method. Protein expression densitometry analysis was performed using Image J 1.52n software. Data are mean ± SEM of three independent experiments. *p < vs NG; ** p < vs HG

As far as autophagy is concerned, HG increased autophagy activity (p < 0.013 vs. NG) while TZT had the opposite effect (p < 0.048 vs HG) (Fig. 7A). HG-induced p62 and Beclin1 mRNA expression and protein levels (p < 0.001vs NG), while the presence of TZT antagonized the HG-related effect (p < 0.001 vs HG) (Fig. 7B, C).

### Exploring TZT's molecular targets: an interactive enrichment approach

**Fig. 8:**
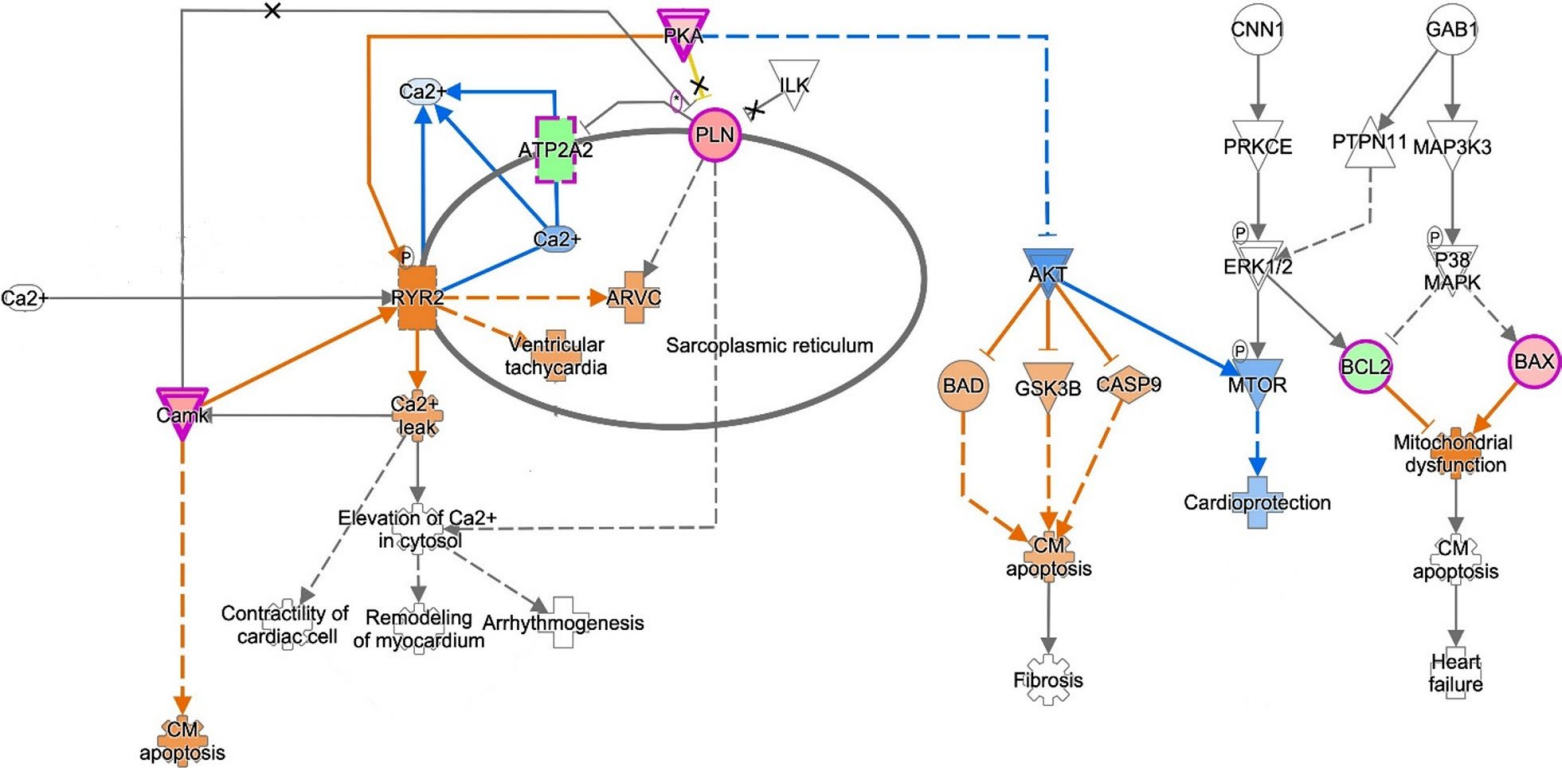
Building interactive models of experimental systems The key entries genes of this work are labeled in larger bold font with red fill. Direct connections between/among genes are shown in solid lines; indirect interactions are shown as dashed lines (also called “edges”). Connections between genes objective of this work are shown in dark blue; interactions between highlighted genes and not directly mapped in this work are shown in turquoise. Target shapes are indicative of function, and the complete legend has been reported in right part of figure

The interactive model, created through Ingenuity Pathway Analysis, confirmed the connections among the analyzed markers and the main pathways involved. It also revealed which mediators impact contractility, remodeling, arrhythmogenesis, apoptosis, and fibrosis, consequently affecting cardiac function (Fig. 8). Having demonstrated in vitro the effects of TZT on all these mediators, this interactive model allows to confirm the identification of the potential and overall mechanism of action of TZT found “in vitro”.

## Discussion

In our study, we explored the cardiac benefits of TZT through three-steps approach. In particular. (i) the meta-analysis showed a reduction in the risk for MACE by TZT; ii) the effects of TZT on cardiac remodeling in human cardiac AC16 cells exposed to high glucose demonstrated that (a) the human cardiac cell line expressed a higher level of GIP receptors than GLP-1 receptors, indicating a greater prevalence of GIP receptors expression in this cell line; (b) TZT, a novel antidiabetic drug with both GIP-Ra and GPL1-Ra activities, displayed a protective cardiac effect preventing cell death, fibrosis and hypertrophy with a potential positive impact on cardiac remodeling; (iii) Ingenuity Pathway Analysis showed an interactive model that confirms the potential mechanism of action of TZT (Graphical abstract).

Heart failure is the most frequent and dangerous cardiac complication in type 2 diabetic patients [20]. Previous data demonstrated that GPL1-RAs are useful for cardiac protection since they reduce heart failure events in type 2 diabetic patients [21]. Liraglutide and semaglutide have been shown to lower the probability of heart failure’s occurrence or worsening (regarding re-hospitalization) by fighting cardiac fibrosis [22–26]. Nevertheless, both drugs are only GPL1-Ras without affecting the GIP receptor. More recently, TZT a new antidiabetic drug displaying both GIP and GLP1 receptor agonist activities, has become commercially available [27].

Despite the evidence that TZT has a powerful impact on metabolic control and an impressive effect on body weight loss [28], data on cardiac protection are scarce.

Interestingly, our meta-analysis indicates that TZT significantly reduced the risk for a MACE compared with control.

Understanding the functional significance of disparity in receptors expression could have significant implications. The heart and blood vessels exhibit expression of GIP and GLP-1 receptors, while the GIP receptors specifically play a prominent role in the ventricular myocardium of both rodents and humans. This widespread expression of GIP receptors strengthens the hypothesis of their direct involvement in modulating cardiac function [14, 29]. In addition, previous results reveal a greater degree of affinity of TZT for the GIP than the GLP-1 receptor [30]. As a whole, such data prompt us to hypothesize the positive cardiac effects of tirzepatide may primarily result from the GIP component and that despite the dual activity on both GIP and GLP1 receptors, the favorable effectiveness of this agent may be attributed to an imbalance favoring the GIP receptor [31].

So far, a TZT-mediated cardiac protective effect cannot be ruled out. Indeed, a previous report indicated that TZT attenuates left ventricular remodeling and dysfunction induced by lipopolysaccharide (LPS) by inhibiting the TLR4/NF-kB/NLRP3 [32]. For such reason, the possibility that tirzepatide might have a role in controlling HG-induced cardiomyocyte death, fibrosis, and hypertrophy cannot be ruled out.

Our findings revealed significant differences in the expression levels of critical genes and proteins involved in hypertrophy and fibrosis in cells exposed to HG in the presence or not of tirzepatide. Indeed, the presence of tirzepatide was associated with a marked inhibition in HG-induced changes in the expression levels of COL1A1, TGF-b, FBXO32, MMP9, MuRF1 and p-Akt.

MMP-9 has received the most attention in cardiac cells due to its mechanical solid connection with cardiac remodeling. Besides ECM constituents, MMP-9 also processes several cytokines and chemokines, including TNFα, IL-1β, TGFβ, and CXC motif ligands (CXCL-1,4,5,7, and 12) [33–35]. Previous studies reported that attenuation of cardiac hypertrophy and fibrosis by liraglutide after angiotensin II infusion is mediated by inhibiting TGF-β1/Smads signaling pathways [36, 37]. Also, research has shown a connection between Atrogin-1/MAFbx and cardiac hypertrophy [38]. MuRF-1 have been identified as essential enzymes in ubiquitin-mediated proteolysis and muscle atrophy, and modulating their expression via physical activity or targeting the upstream cytokines and growth factors that regulate their expression has the potential to prevent or reverse muscle atrophy in patients with sarcopenia [39, 40]. The key mechanism is made up by the acceleration of muscle protein degradation in muscle involving an increase in the expression and activity of two muscle-specific, E3 Ub ligases, Atrogin-1 (also known as MAFbx) and MuRF-1 [34, 41]. Mechanisms that regulate the activation of these E3 Ub-conjugating enzymes have been intensively studied and at least two regulatory factors have been identified, the forkhead transcription factors (FoxO) and the inflammatory transcription factor, NFκB. FoxO and NFκB activate the promoters for Atrogin-1/MAFbx and MuRF-1 respectively and an increase in the expression of these specific E3 Ub ligases stimulates muscle protein degradation in the ubiquitin–proteasome system. Nevertheless, oxidative stress may also play a role since it up-regulates expression of atrogin-1 and MuRF-1 in muscle, and these E3 ligases consequently activate the proteasome [42–44]. The relationship between MuRF1 expression and cardiac fibrosis is complex and not fully understood, and it may vary depending on the specific context, physiological or pathological conditions, and characteristics of the cardiac tissue studied. Indeed, evidence suggests the involvement of E3 ligases, including MuRF1, in regulating cellular processes in the heart, including the response to cardiac fibrosis [45]. Our present data support the direct involvement of MuRF1 in HG-induced cardiac fibrosis, a phenomenon which seems reversed by tirzepatide.

Further support for the involvement of MuRF-1 in cardiac fibrosis came from treatment in H9C2 cell lines by metformin enhanced the activity of MuRF1 and demonstrated a reduction in hypertrophic remodeling [46].

Additionally, our study demonstrated that treatment with Tirzepatide increased the expression and activity of SERCA2 and phosphorylated PLN and decreased the expression of PKA and CAMKII, essential modulators of calcium signaling and cardiac hypertrophy.

As myocardial remodeling plays a crucial role in the development of heart failure and one of the critical characteristics of heart failure is disruptions in the metabolism of cardiac calcium, GLP-1RAs can prevent post-MI remodeling by influencing changes in the extracellular matrix and calcium handling [47, 48]. Previous research showed that elevated intracellular calcium or disruption of its balance can activate CaMKII, causing arrhythmia, heart failure, cardiomyocyte apoptosis, contractile dysfunction, and hypertrophy [49]. Kareusser et al. showed that CaMKII induces maladaptive cardiac remodeling and that its inhibition is a promising approach for attenuating the progression of heart failure [50, 51]. Younce et al. discovered that exendin-4 prevented the decrease in SERCA2 and p-PLN levels in response to hyperglycaemia, indicating a potential influence of exendin-4 on calcium handling and cardiac protective effects [52]. The positive impacts exendin-4 on cardiac remodeling may be attributed to the activation of the eNOS/cGMP/PKG pathway and the inhibition of the CaMKII pathway [53].

We also got evidence that Tirzepatide promoted cell proliferation, as indicated by increased Ki67 expression and enhanced cell viability. Our results demonstrated that the impact of tirzepatide on apoptosis markers revealed a notable decrease in the expression of the pro-apoptotic protein Bax. In contrast, the levels of anti-apoptotic protein Bcl2 exhibited an upregulation. Furthermore, the activation of total and clavated forms of CASP3, a pivotal executioner in apoptotic pathways, was notably attenuated following treatment with tirzepatide. Interestingly, we observed a substantial decrease in the autophagy adaptor proteins p62 and Beclin1, suggesting an enhancement in autophagic flux in response to tirzepatide treatment.

In addition, the creation of an interactive model, driven by the interaction of all markers affected by Tirzepatide, support the potential mechanism of action through which Tirzepatide is able to reduce the risk of heart failure.

We acknowledge that the data obtained using only an in vitro cell system, specifically AC16 cardiac cells, represents a potential limitation of the study. However, obtaining primary human cardiomyocytes poses challenges due to limited availability and viability, as they cannot be maintained in culture for more than a few days. Therefore, after carefully considering these factors, we chose to utilize the AC16 cellular model in our research. Nevertheless, we are aware that it would be desirable to corroborate our findings with further experiments in animal models.

## Conclusions

Our study supports the evidence that Tirzepatide protects against diabetes-related damage to cardiomyocytes. In vitro data support the evidence that Tirzepatide beneficial effect on cardiomyocytes is mediated by a positive modulation of cardiomyocyte death, fibrosis, and hypertrophy occurring in the presence of high glucose concentrations.

### Supplementary Information

**Additional file 1.** Cell viability and toxicity were evaluated in the AC16 cell line exposed to different Tirzepatide (TZT) concentrations in normal (5 mM) and high glucose (33 mM) conditions for seven days. No differences were observed in cell viability and toxicity between different TZT concentrations and control in normal glucose condition (NG) (p > 0.05 vs NG) (**Supplementary Figure 1A**). However, cells exposed to HG for 7 days and treated with TZT at concentrations of 25 nM, and 50 nM showed no difference compared to HG conditions. Moreover, TZT at concentrations of 100 nM, 150 nM, 200 nM, and 250 nM prevented the reduction in cell viability induced by HG (p < 0.05), and the cotreatment with TZT induced a reduction of toxicity compared to HG (p < 0.05) (**Supplementary Figure 1B**).

## Data Availability

The data used and/or analyzed during the current study are available from the corresponding author on reasonable request.

## Abbreviations

GLP-1RA: Glucagon-like peptide-1 receptor agonist
TZT: Tirzepatide
GIP: Glucose-dependent insulinotropic polypeptide
GLP-1R: Glucagon-like peptide-1 receptor
MACE: Major adverse cardiovascular events
HF: Heart failure
LDH: Lactate dehydrogenase
SGLT2: Sodium-glucose co-transporter 2
NG: Normal glucose
HG: High glucose
ECM: Extracellular matrix
MMP9: Matrix metallopeptidase 9
eNOS: Endothelial nitric oxide synthase
cGMP: Cyclic guanosine monophosphate
PKG: Protein kinase G
LPS: Lipopolysaccharide
TLR4: Toll-like receptor 4
NF-kB: Nuclear Factor-kappa B
NLRP3: Nucleotide-Binding Domain, Leucine-Rich–Containing Family, Pyrin Domain–Containing-3
