# Supplementary material for: Evidence that tirzepatide protects against diabetes-related cardiac damages
Source: Cardiovasc Diabetol. 2024 Mar 30;23:112. doi: 10.1186/s12933-024-02203-4 (PMC10981817; doi:10.1186/s12933-024-02203-4)

**Evidence that Tirzepatide Protects Against Diabetes-Related Cardiac Damages**

Fatemeh Taktaz†^a^, Lucia Scisciola†^a^, Rosaria Anna Fontanella^a^, Ada Pesapane^a^, Puja Ghosh^a^, Martina Franzese^a^, Giovanni Tortorella^a^, Armando Puocci^a^, Eduardo Sommella^b^, Giuseppe Signoriello^c^ Fabiola Olivieri ^d,e^, Michelangela Barbieri^a§^ and Giuseppe Paolisso^a,f§^

***^a^*** *Department of Advanced Medical and Surgical Sciences, University of Campania "Luigi Vanvitelli", Naples, Italy.*

***^b^*** *Department of Pharmacy, University of Salerno, Fisciano, SA, Italy.*

***^c^*** *Statistical Unit, Department of Mental Health and Public Medicine, University of Campania, Naples, Italy.*

***^d^*** *Department of Clinical and Molecular Sciences, DISCLIMO, Università Politecnica delle Marche, Ancona, Italy.*

***^e^*** *Center of Clinical Pathology and Innovative Therapy, IRCCS INRCA, Ancona, Italy.*

***^f^*** *UniCamillus, International Medical University, Rome – Italy*

†The authors share the co-first authorship

§ The authors share the co-last authorship

**Address correspondence to:**

Lucia Scisciola, PhD

Tel: ++390815665143

e-mail: [lucia.scisciola@unicampania.it](mailto:lucia.scisciola@unicampania.it)

***Additional File***

Cell viability and toxicity were evaluated in the AC16 cell line exposed to different Tirzepatide (TZT) concentrations in normal (5 mM) and high glucose (33 mM) conditions for seven days. No differences were observed in cell viability and toxicity between different TZT concentrations and control in normal glucose condition (NG) (p> 0.05 vs NG) (Supplementary Figure 1A). However, cells exposed to HG for 7 days and treated with TZT at concentrations of 25 nM, and 50 nM showed no difference compared to HG conditions. Moreover, TZT at concentrations of 100 nM, 150 nM, 200 nM, and 250 nM prevented the reduction in cell viability induced by HG (p<0.05), and the co-treatment with TZT induced a reduction of toxicity compared to HG (p<0.05) (Supplementary Figure 1B).

**Additional file Figure 1**


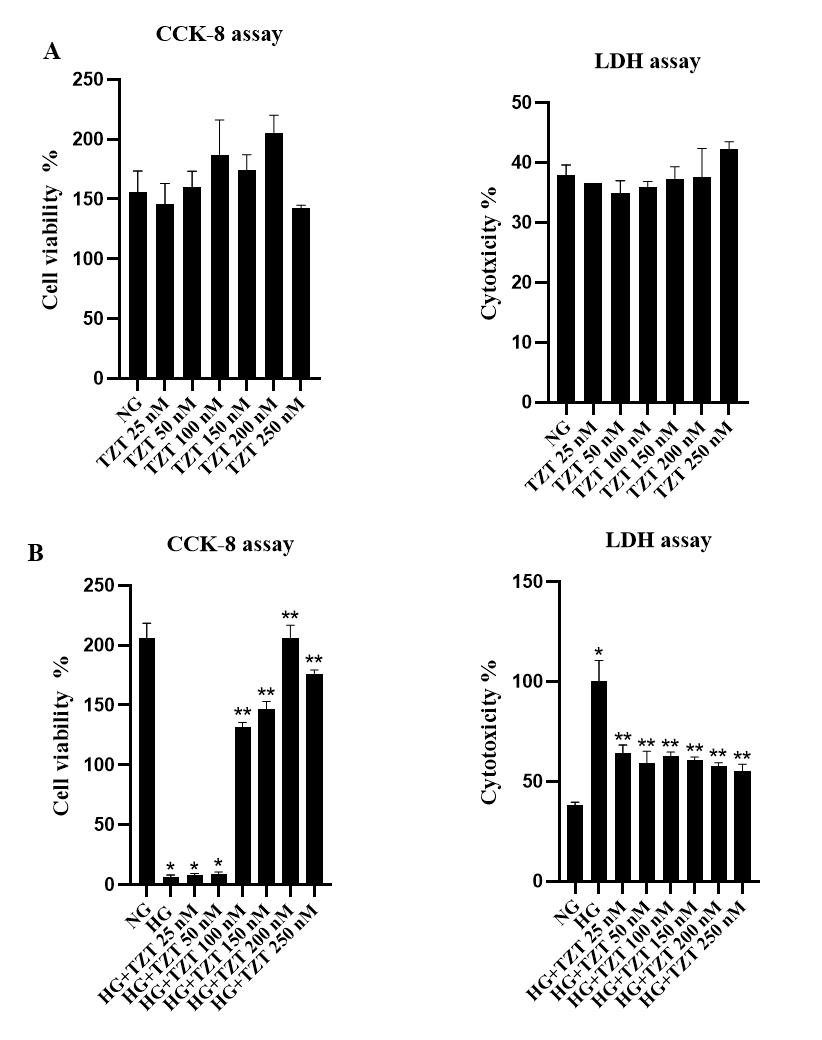

Supplement: Supplementary file 1 — Additional file 1. Cell viability and toxicity were evaluated in the AC16 cell line exposed to different Tirzepatide (TZT) concentrations in normal (5 mM) and high glucose (33 mM) conditions for seven days. No differences were observed in cell viability and toxicity between different TZT concentrations and control in normal glucose condition (NG) (p > 0.05 vs NG) (Supplementary Figure 1A). However, cells exposed to HG for 7 days and treated with TZT at concentrations of 25 nM, and 50 nM showed no difference compared to HG conditions. Moreover, TZT at concentrations of 100 nM, 150 nM, 200 nM, and 250 nM prevented the reduction in cell viability induced by HG (p < 0.05), and the cotreatment with TZT induced a reduction of toxicity compared to HG (p < 0.05) (Supplementary Figure 1B). [file 12933_2024_2203_MOESM1_ESM.docx]
